# Ultra-Hypofractionated Proton Therapy in Localized Prostate Cancer: Passive Scattering versus Intensity-Modulated Proton Therapy

**DOI:** 10.3390/jpm11121311

**Published:** 2021-12-06

**Authors:** Dorota Maria Borowicz, Konstantin N. Shipulin, Gennady V. Mytsin, Agnieszka Skrobała, Piotr Milecki, Victor N. Gayevsky, Vladimir Vondráček, Julian Malicki

**Affiliations:** 1 Greater Poland Cancer Centre, Department of Medical Physics, 61-688 Poznan, Poland; agnieszka.skrobala@wco.pl (A.S.); piotr.milecki@wco.pl (P.M.); julian.malicki@wco.pl (J.M.); 2 Dzhelepov Laboratory of Nuclear Problems, Joint Institute for Nuclear Research, 141980 Dubna, Russia; shipulinkn@yandex.ru (K.N.S.); mytsin@nusun.jinr.ru (G.V.M.); gayevsky@nusun.jinr.ru (V.N.G.); 3 Electroradiology Department, Poznan University of Medical Sciences, 61-688 Poznan, Poland; 4 Greater Poland Cancer Centre, Department of Radiotherapy I-st, 61-886 Poznan, Poland; 5 Proton Therapy Center Czech, 180 00 Prague, Czech Republic; vladimir.vondracek@ptc.cz

**Keywords:** proton therapy, prostate, ultra-hypofractionated radiotherapy

## Abstract

Few studies have directly compared passive scattering (PS) to intensity-modulated proton therapy (IMPT) in the delivery of ultra-hypofractionated proton beams to the localized prostate cancer (PCa). In this preliminary study involving five patients previously treated with CyberKnife, treatment plans were created for PS and IMPT (36.25 CGE in five fractions with two opposing fields) to compare the dosimetric parameters to the planning target volume (PTV) and organs-at-risk (OAR: rectum, bladder, femoral heads). Both plans met the acceptance criteria. Significant differences were observed in the minimum and maximum doses to the PTV. The mean dose to the PTV was lower for PS (35.62 ± 0.26 vs. 37.18 ± 0.14; *p* = 0.002). Target coverage (D98%) was better for IMPT (96.79% vs. 99.10%; *p* = 0.004). IMPT resulted in significantly lower mean doses to the rectum (16.75 CGE vs. 6.88 CGE; *p* = 0.004) and bladder (17.69 CGE vs. 5.98 CGE *p* = 0.002). High dose to the rectum (V36.25 CGE) were lower with PS, but not significantly opposite to high dose to the bladder. No significant differences were observed in mean conformity index values, with a non-significant trend towards higher mean homogeneity index values for PS. Non-significant differences in the gamma index for both fields were observed. These findings suggest that both PS and IMPT ultra-hypofractionated proton therapy for PCa are highly precise, offering good target coverage and sparing of normal tissues and OARs.

## 1. Introduction

Hypofractionated radiotherapy has many advantages over conventional fractionation schemes, including shorter treatment times and more effective cell killing due to the greater sensitivity of prostate cancer cells to high doses per fraction [1,2,3,4,5,6,7,8], which explains the growing interest in this approach to treat a wide range of tumour types, including prostate cancer (PCa) [9].

Stereotactic body radiotherapy (SBRT) is a technique involving the precise delivery of highly conformal, hypofractionated radiation delivered in the form of a photon beam. Highlighted technique is implemented to radiotherapy using a proton beam. Similar schema to SBRT is implemented to radiotherapy using a proton beam. Proton therapy is less common (mainly due to the cost of the equipment), but it has many potential advantages due to the unique physical properties of proton beams, which have a steep fall off (Bragg peak), thus enabling the deposition of very high doses to the target while protecting the organs at risk (OARs).

In recent years, there has been a growing interest in ultra-hypofractionated radiotherapy. This technique involves the administration of large daily fractions (4 to 8 Gy), which can be delivered through either photon or proton beam therapy [10,11,12,13,14,15]. Although both techniques can achieve satisfactory results, proton therapy has a theoretical advantage in terms of better sparing of normal tissues due to dose modulation along the beam path to create a spread-out Bragg peak (SoBP). In proton therapy, there are two main approaches to creating the therapeutic beam, passive scattering (PS) and intensity-modulated proton therapy (IMPT), also known as active pencil beam scanning. Several studies have compared these techniques, without finding any significant differences in terms of late gastrointestinal (GI) or genitourinary (GU) toxicity [16,17,18,19].

At present, only limited data are available for ultra-hypofractionated proton therapy in localized PCa. As a result, more research is needed to investigate the optimal approach for patients with PCa. Therefore, we conducted the present study to compare treatment plan parameters for passive scattering vs. IMPT in a small sample of patients with localized PCa.

## 2. Materials and Methods

### 2.1. Patient Cohort

This was a study involving patients diagnosed with localized PCa treated at our institution (Greater Poland Cancer Centre; GPCC) by CyberKnife. All patients were treated using the SBRT protocol, with a total dose of 36.25 Gy (7.25 Gy/fraction). The patients ranged in age from 69 to 84 years. The prostate volumes were ranged 30–60 cc. Five patients were re-planned using PS and IMPT and these treatment plans were verified in the Medico-Technical Complex at the Joint Institute of Nuclear Research, and in the Czech Proton Therapy Center.

Data from the CT scans were obtained in order to contour the planning target volume (PTV). The planning target volume included the clinical target volume (CTV): the prostate gland plus a margin expanded 5 mm laterally, inferiorly, and superiorly (3 mm posteriorly). OARs included the rectum, bladder, and femoral heads (left and right).

### 2.2. Treatment Plans

A single medical physicist prepared all treatment plans using the same acceptance criteria for both techniques. Two opposing beams (90° and 270°) were used to deliver the proton beam to the prostate gland. The prescription dose to the PTV was 36.25 cobalt gray equivalent (CGE) delivered in five fractions. The total dose was an expression of the relative biological effectiveness (RBE) of the proton beam (1.1) compared to photon beams. The OAR accepted criteria are presented in Table 1.

**Figure 1:**
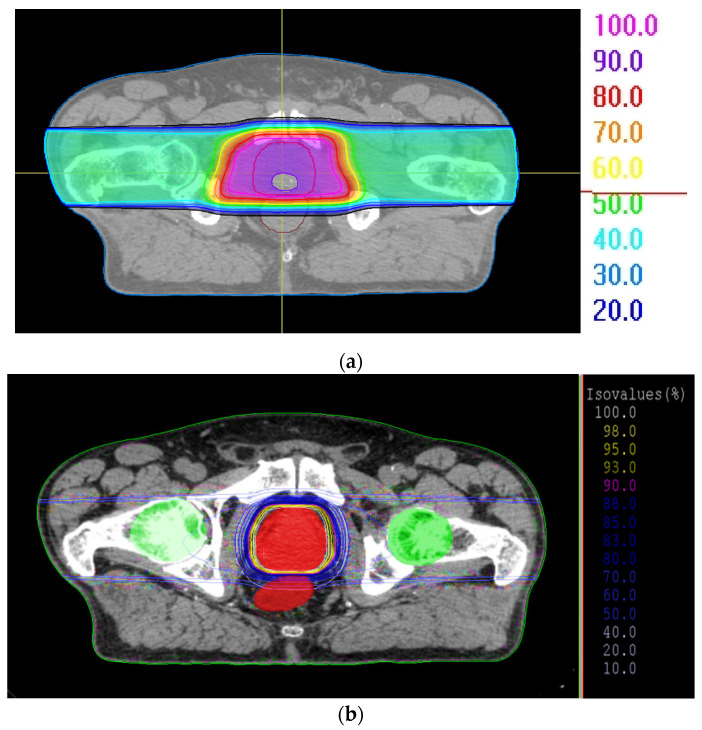
Dose distribution for the same patients (**a**) from RayTreat, and (**b**) with XiO 5.10.

Two proton beam delivery systems and two different treatment planning systems (TPS) were used: RayTreat [20] at the Joint Institute for Nuclear Research (JINR) in Russia, and XiO, release version 5.10 (ELEKTA, Stockholm, Sweden). The first system (JINR Phasotron) was adapted for PS proton beams with output energy of 660 MeV synchrocyclotron. The wide proton beam was delivered by a shaping system composed of a ridge filter, individual collimator, and individual bolus. For the prostate treatment plan in the present study, the energy was reduced to 230 MeV. The field apertures were fitted individually to the PTV in each case. Individual collimators were designed based on the apertures to account for the beam penumbra. For each patient, the aperture of the collimator was cut to protect the rectum the smearing (5 mm), and smoothing margins (9–11 mm) were also calculated for this radiation technique according to the best dose distribution. Margins were chosen individually to calculate a plan which kept the acceptance criteria. For the SOBP determination method, the only one available ridge filter was considered for all patients in cohort. The special prostate ridge filter allowed for wide proton beams covering the entire PTV. Figure 1a shows the TPS interface and dose distribution for a patient.

The second planning system (XiO) was adapted to 235 MeV synchrotron (IBA, Belgium) with active scanning (proton pencil beams). Single field uniform dose (SFUD) enabled delivery of almost exactly half of the dose to the PTV by each field. The spot sigma and spacing among spots were 4 mm in each treatment plan. No additional parameters (beyond the PTV and OAR criteria) were needed to calculate the plan. For promising coverage of PTV and significantly spare OARs, the two-step optimization of the plan was utilized. A SFUD (single-field uniform dose) and a MFO (multi-field optimization) technique was used to optimization a beam size energy dependence. Dose distribution, as calculated in XiO, is shown in Figure 1b.

### 2.3. Treatment Plans: Verification

Five treatment plans were verified in the treatment rooms in both proton centres (using the different proton beam delivery techniques (i.e., PS and IMPT)). The PS-based treatment plans were verified using Gafchromic film EBT-XD (Ashland Inc., Wilmington, DE, USA), lot 08021701, in an I’mRT phantom (IBA, Ottignies-Louvain-la-Neuve, Belgium). Small film pieces were calibrated to obtain the film darkness values corresponding to the adequate dose. Dosimetric calibration of the proton beam was performed according to International Atomic Energy Agency recommendations (TRS-398 protocol). The film was scanned on an Epson Expression 11000 XL scanner (Seiko EPSON Corporation, Nagano, Japan) under the following parameters: transmission mode: 48-bit RGB format; scan resolution: 72 dpi without image correction. The ImageJ v. 2.0 and the OriginPro 2015 software programs (OriginLab Inc., Northampton, MA, USA) were used to analyse data from the films. The dose–response curve was calculated as the function of net optical density to the calibration dose. The formula developed by Devic et al. [21] was used to calculate netOD, as follows:

(1) $$netOD=OD_{unexp}-OD_{exp}=\frac{PV_{unexp}-PV_{bckg}}{PV_{exp}-PV_{bckg}}$$

where *PV_unexp_* and *PV_exp_* are the pixel values for the non-irradiated and irradiated samples, respectively. *PV_bckg_* represents a zero-light transmitted pixel value obtained for the black opaque scan.

The IMPT-based treatment plans were verified using a high-resolution ionisation chamber detector array MatriXX PT (IBA, Ottignies-Louvain-la-Neuve, Belgium) immersed in a water phantom DigiPhant PT (IBA, Belgium). The verification image was registered in OmniPro I’mRT (IBA Dosimetry, Schwarzenbruck, Germany) and compared with data from a TPS for each angle of irradiation separately.

#### Gamma Index

The gamma index (GI) standard method for dose verification was used. The GI was calculated to compare the TPS-calculated doses with their three-dimensional dose distributions meaning that for each point of dose distribution (DD), the value of GI was calculated using dose difference and distance to agreement (DTA) criteria to determine the pass or fail result. Following the methods described elsewhere [22,23], we evaluated the following criteria: distance (depth and width of field: δd = 3 mm) and dose (δ% = 3%), the same standard criteria used in most centres to verify treatment plans.

### 2.4. Data Analysis

All calculated treatment plans were compared according to their PTV parameters (mean, maximum and minimum doses to the PTV). The doses to the OARs (rectum, bladder, and left and right femoral heads) were calculated. Acceptance criteria for doses to the OARs are shown in Table 1. The conformity index (CI) and homogeneity index (HI) were also evaluated. Using the guidelines and recommendations of the International Commission on Radiation Unit and Measurement 83 [24], CI and HI were defined according to the following equations:

(2) $$CI=\frac{V_{RI}}{TV}$$

where *V_RI_* is the volume of prescribed dose for PTV, and *TV* is the total volume of PTV.

(3) $$HI=\frac{I_{max}}{RI}$$

*I_max_* is the maximum dose, and *RI* is the prescribed dose.

Comparison of the GI values of the quantitative variables in the two groups was performed using the Mann–Whitney test, with a cut-off for significance of *p* ≤ 0.05. The statistical analyses were performed using the R software, version 4.0.4 (Vienna, Austria) [25].

## 3. Results

### 3.1. Treatment Plan Parameters—PTV

The PTV parameters obtained in the two treatment plans are shown in Table 2.

**Figure 2:**
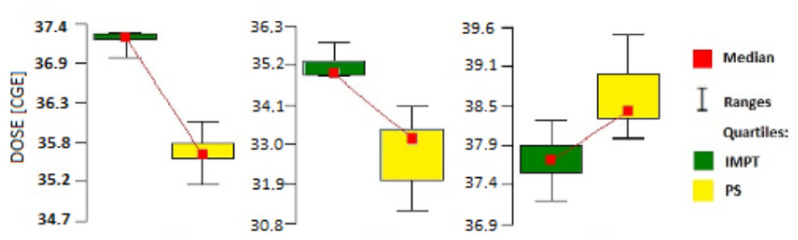
Radiation doses to the PTV according to the technique (PS vs. IMPT): (**a**) mean, (**b**) min, (**c**) max.

As Table 2 shows, there were significant differences between the two techniques. IMPT yielded significantly higher mean and minimum doses. The coverage of PTV by 98% isodose of prescribed dose was better for the IMPT technique as it means that a bigger part of the target received dose was above 98% of 36.25 CGE. By contrast, PS resulted in significantly higher maximum doses to the PTV. Additional data about the PTV doses (median, quartiles and ranges) are shown in Figure 2.

### 3.2. Treatment Plan Parameters for OARs

Table 3 shows the mean dosimetric parameters for OARs based on the treatment plan criteria (Table 1).

#### 3.2.1. Rectum

**Figure 3:**
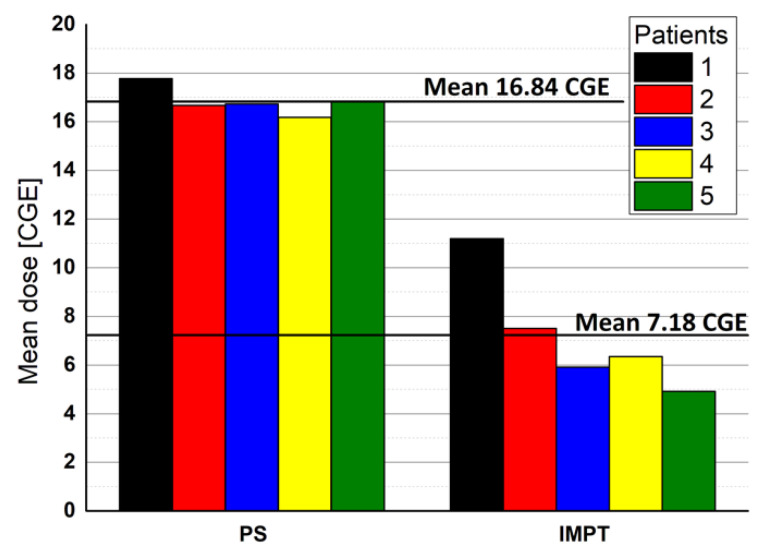
Homogeneity of mean rectal dose for each patient.

As Table 3 shows, there were no significant differences between PS and IMPT in terms of high and low rectal doses, although a trend was observed for lower doses with PS. The mean dose was significant higher with PS, but more homogeneous (Figure 3).

**Figure 4:**
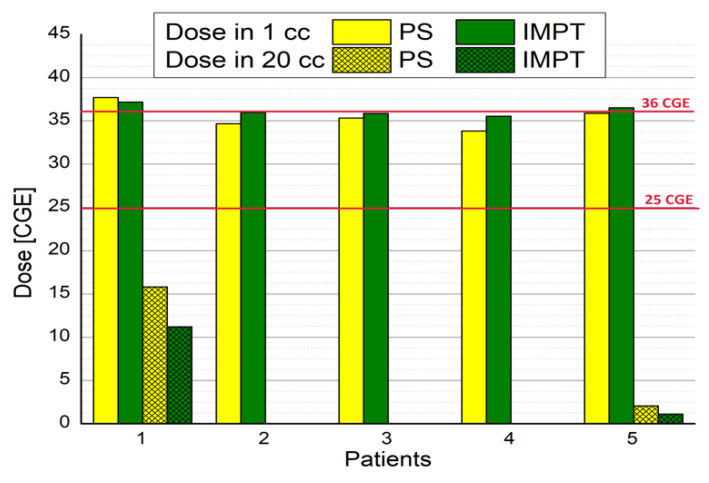
Doses delivered to 1 cc of the rectum for each patient.

The most rigorous dose criteria were satisfied for rectum V36_CGE_ < 1 cc in both techniques: 35.55 CGE vs. 36.19 CGE (*p* = 0.354) for PS and IMPT, respectively. However, this criterion was not met in two patients; consequently, additional criteria from the Czech Proton Therapy Center Czech protocol were considered: rectal dose in 20 cc < 25 CGE. Figure 4 shows the doses delivered to 1 cc of the rectum for each patient in the cohort and the additional criteria for the two exceptions.

#### 3.2.2. Bladder

PS resulted in a significantly higher mean percentage of the bladder volume receiving the low dose (V18 CGE) than IMPT (25.8% vs. 14.58%, *p* = 0.019). However, both techniques met the acceptance criteria (<55% of the bladder). For higher doses (V36.25 CGE), 3.3% of the bladder received this dose with PS vs. only 2.9% with IMPT (*p* = 0.943). The dose delivered to 10 cc was 33.48 CGE for IMPT vs. 34.01 CGE for PS (*p* = 0.509). IMPT delivered significantly lower mean doses to the bladder: 17.69 CGE vs. 5.98 CGE (*p* = 0.002).

#### 3.2.3. Femoral Heads

As expected, both PS and IMPT met the acceptance criteria for the left and right femoral heads. However, PS yielded significantly higher maximum doses to the left and right femoral heads (17.99 CGE and 17.90 CGE) compared to IMPT (14.77 CGE and 14.85 CGE) (*p* = 0.004 for both comparisons).

### 3.3. Treatment Plan Parameters—Conformity and Homogeneity Indices

**Figure 5:**
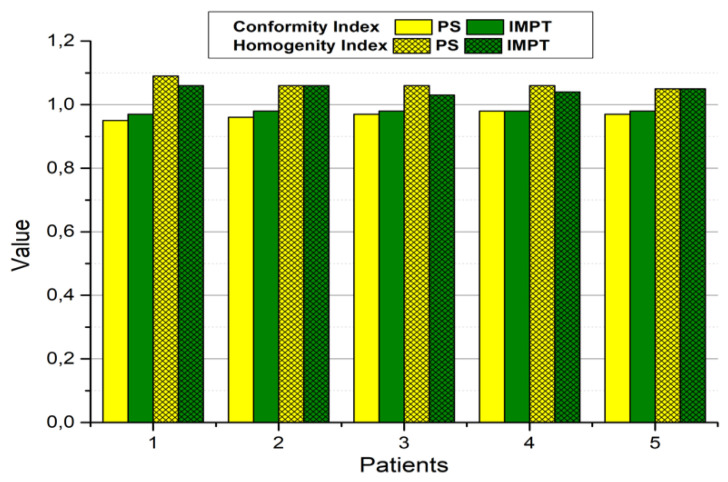
Individual conformity and homogeneity indices for PS and IMPT.

Mean CI values were significantly higher for IMPT (0.98 vs. 0.97; *p* = 0.024), while there was a non-significant trend (*p* = 0.082) for higher HI values with PS than for IMPT (1.06 vs. 1.05). Figure 5 presents the CI and HI values for treatment plans calculated in both techniques for each patient.

### 3.4. Gamma Index

Table 4 shows the GI values calculated separately for each beam field, which were non-significantly lower for PS. In both cases, the GI values were above the 95% acceptance criteria.

## 4. Discussion

The aim of this preliminary study was to compare treatment plan parameters for localized PCa for two ultra-hypofractionated proton beam therapy modalities—passive scattering and IMPT. Both techniques yielded satisfactory treatment plan parameters for the PTV and OARs and, importantly, both also met the acceptance criteria. However, significant differences were observed in the mean, minimum, and maximum doses to the PTV. PS resulted in a significantly lower mean dose to the PTV (35.62 ± 0.26 vs. 37.18 ± 0.14; *p* = 0.002) and worse target coverage (D_98_: 96.79% vs. 100.42%; *p* = 0.004). Mean doses to the rectum (6.88 vs. 16.75 CGE; *p* = 0.004) and bladder (5.98 vs. 17.69 CGE, *p* = 0.002) were significantly lower in IMPT plans. No significant differences were observed in mean CI values, but there was a trend (non-significant) towards higher mean HI values for PS. These findings suggest that both PS and IMPT ultra-hypofractionated proton therapy for localized PCa are highly precise, offering good target coverage and sparing of normal tissues and OARs.

Interest in hypofractionated and ultra-hypofractionated radiotherapy for the treatment of localized PCa continues to grow. Vargas et al. [14] were the first authors to describe an ultra-hypofractionated model for PCa (38 Gy in five fractions). A similar ultra-hyperfractionated technique with IMPT was developed by Kubes et al. [13] and Vargas et al. [14]. Other authors have compared proton beam with PS to photon beam techniques [10,26,27].

Kase et al. [15] compared IMPT and PS using a conventional fractionation scheme, finding that IMPT resulted in better dose density with lower homogeneity to the PTV than PS. That lower homogeneity to the PTV was caused by hotspots near the edge of the PTV, which necessarily arise from the dose contrast between the PTV and the surrounding OARs. Our results confirm that IMPT delivers more accumulated doses to the PTV than PS (Table 2). We also found that IMPT resulted in more homogeneous doses to the PTV with better target coverage (99.10% with IMPT vs. 96.79% with PS). This finding could be due to the need to reach a balance between the optimal PTV and OAR doses. We found that mean PTV doses for both techniques (PS and IMPT, 35.62 and 37.18 CGE, respectively) were lower than those reported by Kole et al. (37.6 CGE) [10].

Using the same fractionation scheme techniques as Kole et al., Moteabbed et al. [5] found that the PTV coverage (D_98_ isodose) was 95%, which is similar to our results (96.79%). The mean PTV dose for IMPT in our study was 37.18 CGE, which is similar to the results observed by Kubes et al. (36.71 CGE) [13]. Compared to standard fractionation, we found a lower mean PTV dose (% prescribed dose) for PS (98.27%) than Vargas et al. [12] (102.4%) and for IMPT when compared to the data reported by Tran et al. (102.6% vs. 104.8%) [28].

Most studies that have compared PS to IMPT in the treatment of localized PCa have evaluated acute and late toxicity. Unfortunately, our study design does not allow us to assess treatment-related toxicity. IMPT is generally considered to have better dose distribution than PS [13,15]. Mishra et al. [17] compared PS to IMPT, finding no differences in mean quality of life scores or acute grade ≥ 2 GI toxicity at one-year, but they did observe higher rates of acute grade ≥ 2 GU toxicity in the IMPT group.

Mean doses to the rectum were higher for PS than for IMPT (16.75 vs. 6.88 CGE); our IMPT result was lower than the dose observed (11.85 CGE) by Kubes et al. [13]. The mean dose to the bladder with IMPT in our study was 5.98 CGE, which compares favourably to the 7.72 CGE reported by Kubes et al. We also observed lower doses for both the left (7.57 vs. 12.06 CGE) and right (8.77 vs. 15.21 CGE) femoral heads compared to those reported by Kubes et al. Similarly, the mean doses for PS were lower than those described by Wessels et al. [26], who retrospectively compared treatment plans for proton, tomotherapy, and CyberKnife, using the treatment plan criteria indicated in RTOG 0938 for SBRT. All of the treatment plans in our study met all RTOG criteria. By contrast, Wessels et al. did not satisfy the high dose criteria for the rectum and bladder (90% of these volumes should receive < 32.6 CGE), which received 33.7 and 36.3 CGE, respectively. That study also exceeded the limits for the bladder V50% (<18 CGE), which received 20.30 CGE. By contrast, the V50 for the rectum (19.10 CGE) was well under the limits.

In our study, the mean doses to the OARs for PS were higher than those reported by Kole et al. [10] (rectum: 16.75 vs. 6.66 CGE, and bladder: 17.69 vs. 13.7 CGE). For the femoral heads, our results were comparable to those reported by Kole et al.: 11.59 vs. 10.4 CGE (left head) and 12.23 vs. 10.9 CGE (right head). These small differences could be attributed to the number of patients in the experimental cohort and anatomical differences (i.e., PTV and OAR size and proximity to abdominal organs).

For the mean PTV dose, we observed significant differences between PS and IMPT. IMPT provided better PTV coverage, with a homogeneous dose that was closer to the prescribed dose than PS (100.42% vs. 96.79%). The better conformity achieved with IMPT is due to the fact that the PTV is cut into equal slices and each slice is “painted spot by spot” with the precalculated dose, which leads to (non-significantly) higher CI values vs. PS (0.98 vs. 0.97).

In PS, the SOBP was obtained by the stationary ridge filter for each patient; thus, depending on the size of the prostate, this dose homogeneity could vary, with a prescribed dose observed in front of and behind the PTV. Consequently, the HI was higher for PS (1.06 vs. 1.05, *p* = 0.082). Non-significant differences were observed for the dose criteria to OARs, with PS yielding better results for the rectum and worse for the bladder (Table 3). In two patients, the most restrictive criteria (V36_CGE_ < 1 cc) were not met. Consequently, for those two patients, we included an additional criterion from the Proton Therapy Center (PTC) protocol (rectal dose in 20 cc < 25 CGE), which was met. The mean dose for IMPT was lower for all OARs, suggesting that IMPT should result in less GI and GU toxicity [16,17,18,19].

With regards to GI toxicity, both PS and IMPT met the 95% acceptance for the distance (depth and width of field, δd = 3 mm) and dose criteria (δ% = 3%). For PS, the first field—90°—was 98.56%, and the second—270°—was 96.58% (vs. 100% for both fields in IMPT). Unfortunately, due to the lack of published data, we cannot compare these findings to other studies.

### Study Strengths and Limitations

The main limitation of this study is the small sample size, which reflects the preliminary nature of the study. By contrast, an important strength is that this study is—to our knowledge—the first to directly compare PS and IMPT ultra-hypofractionated proton therapy for localized PCa, providing dosimetric results with verification of the calculated treatment plans.

## 5. Conclusions

The present study provides a preliminary comparison of two different proton beam delivery techniques (passive scattering and IMPT) for localized PCa, using an ultra-hypofractionated dose of 7.25 CGE. Compared to IMPT, dose homogeneity to the prostate and OARs was significantly worse with passive scattering. Moreover, IMPT achieved significantly lower mean doses to specific OARs. However, both techniques met all acceptance criteria and spared normal tissues and OARs.

## Figures and Tables

**Table 1:** Treatment plan acceptance criteria for organs-at-risk (OARs).

Rectum	Bladder	Femoral Head (Left and Right)
V18 CGE < 50% Vol	V18 CGE < 55% Vol	V25 CGE < 45% Vol
V29 CGE < 20% Vol	V29 CGE < 25% Vol	
V32.5 CGE < 10% Vol	V32.5 CGE < 15% Vol	
V36.25 CGE < 5% Vol	V36.25 CGE < 10% Vol	
V36 CGE < 1 cc	V37 CGE < 10cc	

Abbreviations: CGE, cobalt gray equivalent; Vol, volume; cc, cubic centimeter.

**Table 2:** PTV dosimetric parameters for passive scattering and intensity modulated proton therapy.

	PS	IMPT	*p*
Mean dose to PTV (CGE)	35.62 ± 0.26	37.18 ± 0.14	*p* = 0.002
Minimum dose to PTV (CGE)	32.75 ± 1.11	35.19 ± 0.41	*p* = 0.004
Maximum dose to PTV (CGE)	38.63 ± 0.57	37.75 ± 0.41	*p* = 0.01
Target coverage D98% (%)	96.79 ± 0.81	99.10 ± 0.10	*p* = 0.004

Abbreviations: PTV, planning target volume; PS, passive scattering; IMPT, intensity-modulated proton therapy.

**Table 3:** Mean value of dosimetric parameters for OARs for PS and IMPT.

	PS	IMPT	*p*
**Rectum**			
V18_CGE_	21.32 ± 5.11 %	17.36 ± 6.23 %	*p* = 0.093
V29_CGE_	9.59 ± 3.1 %	8.99 ± 3.72 %	*p* = 0.524
V32.5_CGE_	5.55 ± 2.27 %	5.88 ± 2.81 %	*p* = 0.943
V36.25_CGE_	1.25 ± 1.18 %	1.93 ± 1.52 %	*p* = 0.171
Dose at vol = 1 cc (<36 CGE)	35.55 ± 1.16 CGE	36.19 ± 0.65 CGE	*p* = 0.354
Mean dose	16.75 ± 0.47 CGE	6.88 ± 2.43 CGE	*p* = 0.004 *
Maximum dose	37.61 ± 0.61 CGE	37.50 ± 0.42 CGE	*p* = 1
**Bladder**			
V18_CGE_	25.8 ± 7.37 %	14.58 ± 5.95 %	*p* = 0.019 *
V29_CGE_	13.74 ± 4.65 %	8.35 ± 3.35 %	*p* = 0.03 *
V32.5_CGE_	9.67 ± 3.62 %	6.01 ± 2.3 %	*p* = 0.065
V36.25_CGE_	3.3 ± 1.6 %	2.9 ± 1.12 %	*p* = 0.943
Dose at vol = 10 cc (<37 CGE)	34.01 ± 2.26 CGE	33.48 ± 1.66 CGE	*p* = 0.509
Mean dose	17.69 ± 0.42 CGE	5.98 ± 2.33 CGE	*p* = 0.002 *
Maximum dose	37.74 ± 0.45 CGE	37.55 ± 0.24 CGE	*p* = 0.824
**Left femoral head**			
V25_CGE_	0	0	
Mean dose	11.59 ± 2.10 CGE	7.57 ± 1.25 CGE	*p* = 0.007 *
Maximum dose	17.99 ± 0.54 CGE	14.72 ± 0.29 CGE	*p* = 0.004 *
**Right femoral head**			
V25_CGE_	0	0	
Mean dose	12.23 ± 2.62 CGE	8.77 ± 1.60 CGE	*p* = 0.006 *
Maximum dose	17.90 ± 0.57 CGE	14.85 ± 0.22 CGE	*p* = 0.004 *

* *p* values < 0.05 were considered significant. Abbreviations: PTV, planning target volume; OAR, organs at risk; PS, passive scattering; IMPT, intensity-modulated proton therapy.

**Table 4:** Gamma index comparison of PS and IMPT.

Mean Value	PS	IMPT	*p*
1st field (90°)	98.56	100	*p* = 0.319
2nd field (270°)	96.58	100	*p* = 0.085

## Data Availability

All detailed data included in the study are available upon appropriate request by contact with the corresponding author.
